# Early administration of norepinephrine increases cardiac preload and cardiac output in septic patients with life-threatening hypotension

**DOI:** 10.1186/cc9207

**Published:** 2010-07-29

**Authors:** Olfa Hamzaoui, Jean-François Georger, Xavier Monnet, Hatem Ksouri, Julien Maizel, Christian Richard, Jean-Louis Teboul

**Affiliations:** 1 Service de réanimation médicale, Centre Hospitalo-Universitaire de Bicêtre, Assistance Publique-Hôpitaux de Paris, EA 4046, Université Paris Sud, 78 rue du Général Leclerc, 94 270 Le Kremlin-Bicêtre, France

## Abstract

**Introduction:**

We sought to examine the cardiac consequences of early administration of norepinephrine in severely hypotensive sepsis patients hospitalized in a medical intensive care unit of a university hospital.

**Methods:**

We included 105 septic-shock patients who already had received volume resuscitation. All received norepinephrine early because of life-threatening hypotension and the need to achieve a sufficient perfusion pressure rapidly and to maintain adequate flow. We analyzed the changes in transpulmonary thermodilution variables associated with the increase in mean arterial pressure (MAP) induced by norepinephrine when the achieved MAP was ≥65 mm Hg.

**Results:**

Norepinephrine significantly increased MAP from 54 ± 8 to 76 ± 9 mm Hg, cardiac index (CI) from 3.2 ± 1.0 to 3.6 ± 1.1 L/min/m^2^, stroke volume index (SVI) from 34 ± 12 to 39 ± 13 ml/m^2^, global end-diastolic volume index (GEDVI) from 694 ± 148 to 742 ± 168 ml/m^2^, and cardiac function index (CFI) from 4.7 ± 1.5 to 5.0 ± 1.6 per min. Beneficial hemodynamic effects on CI, SVI, GEDVI, and CFI were observed in the group of 71 patients with a baseline echocardiographic left ventricular ejection fraction (LVEF) >45%, as well as in the group of 34 patients with a baseline LVEF ≤45%. No change in CI, SVI, GEDVI, or CFI was observed in the 17 patients with baseline LVEF ≤45% for whom values of MAP ≥75 mm Hg were achieved with norepinephrine.

**Conclusions:**

Early administration of norepinephrine aimed at rapidly achieving a sufficient perfusion pressure in severely hypotensive septic-shock patients is able to increase cardiac output through an increase in cardiac preload and cardiac contractility. This effect remained in patients with poor cardiac contractility except when values of MAP ≥75 mm Hg were achieved.

## Introduction

International recommendations have placed either norepinephrine or dopamine as a first-line vasopressor during septic shock [1]. Norepinephrine is considered more potent [1,2] and induces fewer adverse effects than dopamine [3]. However, the effects of norepinephrine on cardiac output and heart function of sepsis patients are uncertain. Norepinephrine can increase cardiac output through several mechanisms: (a) improvement of the left ventricular function explained by two effects: direct β_1_-agonist inotropic effect and α-agonist-mediated restoration of diastolic arterial pressure that represents the driving pressure for coronary perfusion of the left heart; (b) increase in cardiac preload secondary to the α-agonist-mediated decrease in systemic venous capacitance [4]. Conversely, norepinephrine could decrease cardiac output owing to an increase in left ventricular afterload. This latter effect is assumed to be significant if the left ventricular function is already impaired or if an excessive mean arterial pressure (MAP) is targeted, or both. Variable results were reported in studies examining the effects of norepinephrine on cardiac output in septic-shock patients [2,5-12]. This variability can be ascribed to differences in the underlying cardiac function, or the level of MAP actually achieved, or the degree of prior preload optimization or a combination of these. In most previous studies [2,5,9,11], the cardiac output was not altered by norepinephrine, but the drug was administered under conditions of a hyperdynamic state, generally after abundant fluid administration. Because of the recent emphasis on urgent and aggressive resuscitation as a key issue in the management of septic shock [13], current international guidelines recommend to administer a vasopressor early to sustain life and maintain perfusion in the face of life-threatening hypotension, even when hypovolemia has not yet been resolved [1]. We hypothesized that under such conditions, norepinephrine is capable of increasing cardiac output through an increase in cardiac preload. We aimed at checking this hypothesis in an observational study performed in a series of septic-shock patients who early received norepinephrine to maintain perfusion in the face of life-threatening hypotension [1]. We paid particular attention to patients with prior left ventricular dysfunction and particularly when a high MAP value was achieved.

## Materials and methods

### Patients

This was an observational study undertaken over a period of 16 months in patients admitted into the medical intensive care unit (ICU) of the Bicêtre University Hospital for septic shock, as defined by the Surviving Sepsis Campaign [1]. The study was approved by the Institutional Review Board of our institution (Comité pour la protection des personnes de l'Hôpital de Bicêtre), and all patients or their relatives gave their informed consent for the patient's data to be used in the analysis.

The patient's data were included in the analysis (a) if the patients were admitted into the ICU for less than 6 hours, and (b) when the MAP was lower than 65 mm Hg and the attending physician considered it necessary to introduce norepinephrine or increase its dose with the goal of increasing the MAP to above 65 mm Hg. In line with the current recommendations [1], our physicians are used to introducing norepinephrine (or increasing its dose) early in the case of life-threatening hypotension, regardless the degree of prior volume resuscitation and in particular if the diastolic blood pressure remains low (≤40 mm Hg or more in case of prior hypertension) [14]. Another criterion of inclusion was that a PiCCOplus monitor (Pulsion Medical Systems, Munich, Germany) was already in place for routine hemodynamic monitoring.

The criteria of exclusion were the need for simultaneously administering other vasoactive drugs or a new fluid challenge or blood transfusion and the need for modifying the ventilatory settings or the dose of the sedative drugs.

### Hemodynamic variables

The PiCCOplus device allowed continuous measuring of the arterial pressure and the heart rate through a femoral arterial catheter. Each time the attending physician decided to perform any therapeutic intervention such as introduction of norepinephrine or increase in its doses, a series of three 15-ml saline solution boluses were performed through the central venous access before and after the intervention. The average of the three values of transpulmonary thermodilution cardiac index (CI) was then recorded. Other transpulmonary thermodilution variables were recorded: the stroke volume index (SVI) calculated as CI/heart rate, the global end-diastolic volume index (GEDVI) assumed to be a global cardiac preload indicator [15,16], and the cardiac function index (CFI), considered an indicator of the systolic cardiac function [17-19]. An index of systemic vascular resistance (SVRI) was calculated as MAP/CI. The stroke-volume variation (SVV) automatically calculated by the PiCCOplus monitor, was collected in the mechanically ventilated patients who were fully adapted to their ventilator and who did not exhibit cardiac arrhythmias.

Because we are used to performing transthoracic echocardiography (EnVisor Philips B.0; Philips Medical System, Andover, MA) in patients with shock as soon as possible after their admission, we were able to collect baseline measurements of the left ventricular ejection fraction (LVEF) by using the Simpson's method from the apical two- and four-chamber views.

### Data analysis

We analyzed the hemodynamic variables recorded at two consecutive time points: before and after either introduction of norepinephrine or increase in its dose, while the achieved value of MAP was ≥65 mm Hg. The time between the two consecutive measurements did not exceed 2 hours.

We subdivided our population into two groups in function of the median value of MAP achieved with norepinephrine at the second time point. We then separately analyzed the hemodynamic variables depending on whether the median value of MAP was reached.

We also subdivided the whole population into two groups in function of the existence of altered LVEF at baseline, defined by a threshold value of 45%. We then examined the hemodynamic consequences of norepinephrine in patients with LVEF >45% and in patients with LVEF ≤45%. In these two subgroups, we also calculated the median value of MAP reached with norepinephrine at the second time point and then separately analyzed the hemodynamic variables depending on whether the median value of MAP was reached.

### Statistical analysis

All data were normally distributed (Kolmogorov-Smirnov test for normality), except the dose of norepinephrine. The results are expressed as the mean ± SD or as the median (25th to 75th percentile), as appropriate. The statistical comparisons were made by using the paired Student *t *test or the Wilcoxon test, as appropriate. A *P *value of 0.05 was considered statistically significant. All the statistical comparisons were carried out by using Medcalc 9.3.1.0 (Mariakerke, Belgium).

## Results

We included 105 patients within the first 6 hours of their admission in the ICU. Their general characteristics are presented in Table 1. At baseline, the diastolic arterial pressure was ≤40 mm Hg in 67 patients, between 41 and 48 mm Hg in 30 patients (70% of them had a history of hypertension), and between 49 and 55 mm Hg in eight patients (all of them had a history of hypertension).

**Table 1:** General patients' characteristics

	Values
Number of patients	105
Age, years	63 ± 12
Sex ratio, male/female	71/34
Mechanically ventilated patients, %	86
Baseline plasma lactate concentration, mmol/L	3.8 ± 1.2
SOFA	12 ± 2
SAPS II	57 ± 12
Prior hypertension, *n*	29
Prior cardiomyopathy, *n*	30
Baseline left ventricular ejection fraction, %	49 ± 10
Origin of sepsis	
Lung, *n*	83
Abdomen, *n*	10
Urinary tract, *n*	8
Others, *n*	4

The median volume of saline infused in our ICU before inclusion in the study was of 1,000 [500 to 1,500] ml. The median interval between two time points was 60 (30 to 90) minutes.

Before inclusion, 57 patients were already receiving norepinephrine at a dose ineffective to achieve a MAP >65 mm Hg (or >75 mm Hg in cases of prior hypertension). In these patients, the dose of norepinephrine was increased from 0.40 (0.24 to 0.59) μg/kg/min to 0.60 (0.47 to 0.95) μg/kg/min (*P *< 0.001). In the remaining 48 patients, norepinephrine was introduced at the time of the study. Its dose reached a median value of 0.24 (0.18 to 0.47) μg/kg/min at the second time point. Patients did not receive other drugs, except antibiotics (for all of them) and sedative drugs for those receiving mechanical ventilation (propofol alone or in combination with fentanyl or remifentanyl).

### Effects of norepinephrine in the whole population

**Figure 1:**
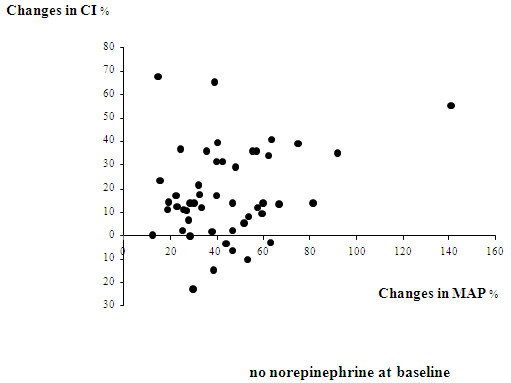
**Relative percentage changes in the cardiac index (CI) and the mean arterial pressure (MAP) before and after introduction of norepinephrine in the subgroup of 48 patients with no norepinephrine at baseline**. The arrows indicate the directions of these changes.

**Figure 2:**
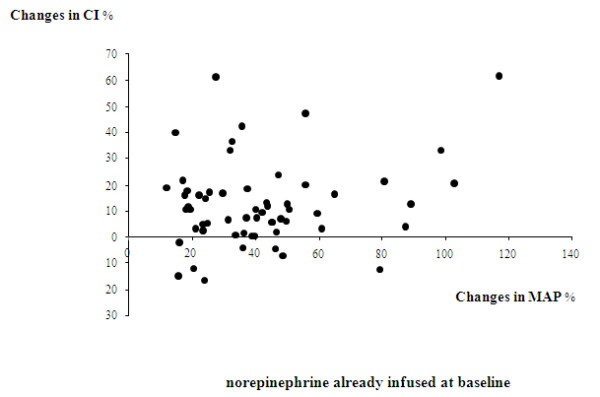
**Relative percentage changes in the cardiac index (CI) and the mean arterial pressure (MAP) before and after increase in the dose of norepinephrine in the subgroup of 57 patients who already received norepinephrine at baseline**. The arrows indicate the directions of these changes.

Norepinephrine significantly increased CI, SVI, GEDVI, and CFI in the global population (Table 2). The significant increases in CI, SVI, GEDVI, and CFI were found in the subgroup of 48 patients in whom norepinephrine was introduced, as well as in the subgroup of 57 patients in whom the dose of norepinephrine was increased (data not presented). Figures 1 and 2 present the percentage changes in CI and MAP before and after norepinephrine introduction/increase in these two subgroups.

**Table 2:** Hemodynamic variables before and after introduction of norepinephrine (or increase in its dose) in the whole population (*n *= 105)

	Before norepinephrine (introduction/increase)	After norepinephrine (introduction/increase)
Heart rate, beats/min	98 ± 21	97 ± 19
MAP, mm Hg	54 ± 8	76 ± 9^a^
DAP, mm Hg	38 ± 6	52 ± 8^a^
CI, L/min/m^2^	3.2 ± 1.0	3.6 ± 1.1^a^
SVI, ml/m^2^	34 ± 12	39 ± 13^a^
GEDVI, ml/m^2^	694 ± 148	742 ± 168^a^
CFI, per minute	4.7 ± 1.5	5.0 ± 1.6^a^
SVRI, dynes/sec/cm^5^/m^2^	1,471 ± 481	1,822 ± 502^a^

CFI, cardiac function index; CI, cardiac index; DAP, diastolic arterial pressure; GEDVI, global end-diastolic volume index; HR, heart rate; MAP, mean arterial pressure; SVI, stroke volume index; SVRI, index of systemic vascular resistance.

^a^*P *< 0.05 vs. before norepinephrine.

In the 59 mechanically ventilated patients (tidal volume, 6.7 ± 1.0 ml/kg) in whom the SVV was recorded, norepinephrine introduction/increase significantly decreased the SVV from 13 ± 6% to 9 ± 5%. In this subgroup, introduction/increase of norepinephrine also significantly increased CI, SVI, GEDVI, and CFI (data not presented).

**Figure 3:**
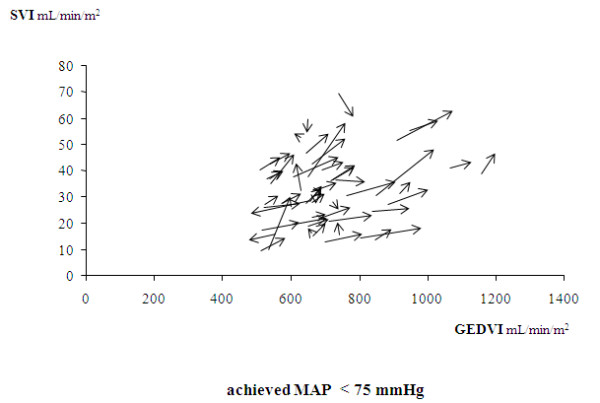
**Relative changes in the individual values of the stroke volume index (SVI) and of the end-diastolic volume index (GEDVI) before and after norepinephrine introduction (or increase in its doses) in the subgroup of 53 patients with an achieved value MAP value <75 mm Hg**. The arrows indicate the directions of these changes.

**Figure 4:**
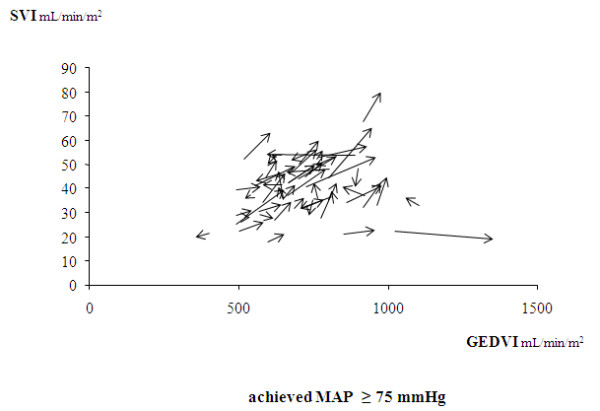
**Relative changes in the individual values of the stroke volume index (SVI) and of the end-diastolic volume index (GEDVI) before and after norepinephrine introduction (or increase in its doses) in the subgroup of 52 patients with an achieved MAP value of ≥75 mm Hg**. The arrows indicate the directions of these changes.

The median MAP value achieved after norepinephrine introduction/increase was 75 mm Hg. The achieved MAP was >85 mm Hg in 16% of patients. The increasing effects of norepinephrine on CI, SVI, GEDVI, and CFI were found in the subgroup of 53 patients with an achieved value of MAP <75 mm Hg, as well as in the subgroup of 52 patients with an achieved value of MAP of ≥75 mm Hg (Table 3). Figures 3 and 4 present the individual changes in SVI and GEDVI in the two subgroups.

**Table 3:** Hemodynamic variables in the whole population depending on whether the achieved mean arterial pressure after introduction of norepinephrine (or increase in its dose) was either inferior (*n *= 53) or superior (*n *= 52) to the median value (75 mm Hg)

	Achieved MAP <75 mm Hg		Achieved MAP ≥75 mm Hg	
		
	Before NE (introduction/increase)	After NE (introduction/increase)	Before NE (introduction/increase)	After NE (introduction/increase)
Heart rate, beats/min	103 ± 20	101 ± 20	93 ± 21	94 ± 20
MAP, mm Hg	51 ± 7	68 ± 4^a^	57 ± 7	83 ± 6^a^
DAP, mm Hg	36 ± 7	48 ± 7^a^	40 ± 6	56 ± 8^a^
CI, L/min/m^2^	3.2 ± 1.1	3.6 ± 1.1^a^	3.2 ± 0.9	3.7 ± 1.1^a^
SVI, ml/m^2^	32 ± 12	37 ± 13^a^	38 ± 12	42 ± 14^a^
GEDVI, ml/m^2^	698 ± 139	752 ± 161^a^	691 ± 157	743 ± 181^a^
CFI, per min	4.7 ± 1.8	4.9 ± 1.7^a^	4.8 ± 1.3	5.1 ± 1.5^a^
SVRI, dynes/sec/cm^5^/m^2^	1,435 ± 535	1,704 ± 592^a^	1,497 ± 542	1,937 ± 582^a^

CFI, cardiac function index; CI, cardiac index; DAP, diastolic arterial pressure; GEDVI, global end-diastolic volume index; HR, heart rate; MAP, mean arterial pressure; NE, norepinephrine; SVI, stroke volume index; SVRI, index of systemic vascular resistance.

^a^*P *< 0.05 vs. before norepinephrine.

### Effects of norepinephrine in patients with a baseline LVEF >45%

**Figure 5:**
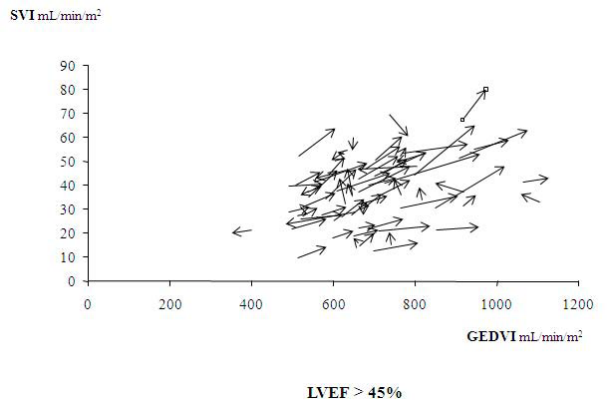
**Relative changes in the individual values of the stroke volume index (SVI) and of the end-diastolic volume index (GEDVI) before and after norepinephrine introduction (or increase in its doses) in the subgroup of 71 patients with a left ventricular ejection fraction (LVEF) >45%**. The arrows indicate the directions of these changes.

Seventy-one patients had a baseline LVEF >45% (median LVEF, 55(50 to 60)%). In this population, norepinephrine significantly increased CI, SVI, and GEDVI (Table 4). Figure 5 presents the individual changes in SVI and GEDVI in this subgroup.

**Table 4:** Hemodynamic variables depending on whether the baseline value of the left ventricular function was either ≤45% (*n *= 34) or >45% (*n *= 71)

	LVEF ≤45%		LVEF >45%	
		
	Before NE (introduction/increase)	After NE (introduction/increase)	Before NE (introduction/increase)	After NE (introduction/increase)
Heart rate, beats/min	96 ± 17	94 ± 17	93 ± 21	94 ± 20
MAP, mm Hg	54 ± 9	75 ± 9^a^	54 ± 7	76 ± 9^a^
DAP, mm Hg	38 ± 7	52 ± 9^a^	38 ± 6	52 ± 8^a^
CI, L/min/m^2^	2.9 ± 0.9	3.3 ± 0.9^a^	3.2 ± 0.9	3.7 ± 1.1^a^
SVI, ml/m^2^	32 ± 11	35 ± 12^a^	36 ± 13	41 ± 15^a^
GEDVI, ml/m^2^	719 ± 161	768 ± 190^a^	683 ± 140	731 ± 157^a^
CFI, per min	4.2 ± 1.4	4.4 ± 1.5	5.0 ± 1.6	5.4 ± 1.6^a^
SVRI, dynes/sec/cm^5^/m^2^	1,588 ± 513	2,024 ± 616^a^	1,435 ± 484	1,680 ± 535^a^

CFI, cardiac function index; CI, cardiac index; DAP, diastolic arterial pressure; GEDVI, global end-diastolic volume index; HR, heart rate; LVEF, left ventricular function; MAP, mean arterial pressure; NE, norepinephrine; SVI, stroke volume index; SVRI, index of systemic vascular resistance.

^a^*P *< 0.05 vs. before norepinephrine.

When subdividing this group of patients by the function of their median value (75 mm Hg), these effects were found in the subgroup of 36 patients with an achieved MAP <75 mm Hg, as well as in the subgroup of 35 patients with an achieved value ≥75 mm Hg (Table 5).

**Table 5:** Hemodynamic variables in the subgroups of patients with left ventricular ejection fraction >45% and an achieved mean arterial pressure either inferior (*n *= 36) or superior (*n *= 35) to the median value (75 mm Hg)

	Achieved MAP <75 mm Hg		Achieved MAP ≥75 mm Hg	
		
	Before NE (introduction/increase)	After NE (introduction/increase)	Before NE (introduction/increase)	After NE (introduction/increase)
Heart rate, beats/min	105 ± 22	104 ± 21	93 ± 23	95 ± 21
MAP, mm Hg	52 ± 7	69 ± 5^a^	56 ± 6	83 ± 6^a^
DAP, mm Hg	37 ± 7	48 ± 7^a^	39 ± 4	56 ± 8^a^
CI, L/min/m^2^	3.2 ± 1.1	3.6 ± 1.1^a^	3.5 ± 1.1	4.1 ± 1.3^a^
SVI, ml/m^2^	32 ± 13	36 ± 13^a^	40 ± 12	46 ± 15^a^
GEDVI, ml/m^2^	674 ± 133	731 ± 157^a^	687 ± 147	729 ± 160^a^
CFI, per min	4.8 ± 1.7	5.0 ± 1.6	5.3 ± 1.7	5.8 ± 1.7^a^
SVRI, dynes/sec/cm^5^/m^2^	1,435 ± 487	1,639 ± 486^a^	1,405 ± 503	1,725 ± 577^a^

CFI, cardiac function index; CI, cardiac index; DAP, diastolic arterial pressure; GEDVI, global end-diastolic volume index; HR, heart rate; MAP, mean arterial pressure; NE, norepinephrine; SVI, stroke volume index; SVRI, index of systemic vascular resistance.

^a^*P *< 0.05 vs. before norepinephrine.

### Effects of norepinephrine in patients with a baseline LVEF ≤45%

**Figure 6:**
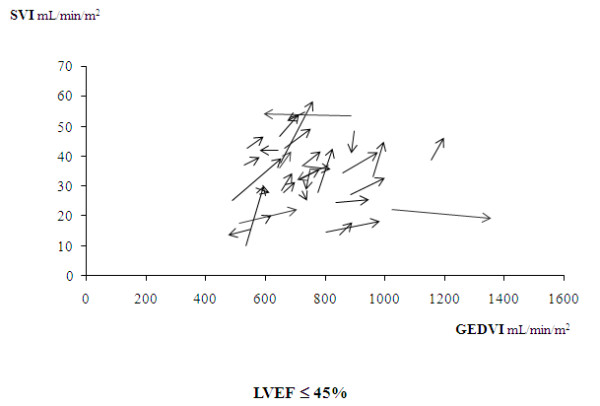
**Relative changes in the individual values of the stroke volume index (SVI) and of the end-diastolic volume index (GEDVI) before and after norepinephrine introduction (or increase in its doses) in the subgroup of 34 patients with a left ventricular ejection fraction (LVEF) ≤45%**. The arrows indicate the directions of these changes.

Thirty-four patients had a baseline LVEF ≤45% (mean LVEF, 40(35 to 40)%). In this population, norepinephrine significantly increased CI, SVI, and GEDVI (Table 4). Figure 6 presents the individual changes in SVI and GEDVI in this subgroup.

When subdividing this group of patients in function of their median value (75 mm Hg), these effects were found in the subgroup of 17 patients with an achieved MAP <75 mm Hg. However, CI, SVI, and GEDVI did not change in the subgroup of 17 patients with an achieved value ≥75 mm Hg (Table 6).

**Table 6:** Hemodynamic variables in the subgroups of patients with left ventricular ejection fraction ≤45% and an achieved mean arterial pressure inferior (*n *= 17) or superior (*n *= 17) to the median value (74 mm Hg)

	Achieved MAP < 75 mm Hg		Achieved MAP ≥75 mm Hg	
		
	Before NE (introduction/increase)	After NE (introduction/increase)	Before NE (introduction/increase)	After NE (introduction/increase)
Heart rate, beats/min	100 ± 16	96 ± 17	92 ± 18	91 ± 17
MAP, mm Hg	49 ± 8	68 ± 4^a^	58 ± 8	82 ± 7^a^
DAP, mm Hg	36 ± 6	48 ± 7^a^	40 ± 8	56 ± 9^a^
CI, L/min/m^2^	2.7 ± 0.9	3.0 ± 1.0^a^	3.2 ± 0.8	3.4 ± 0.8
SVI, ml/m^2^	28 ± 11	33 ± 13^a^	35 ± 10	38 ± 10
GEDVI, ml/m^2^	660 ± 167	770 ± 185^a^	737 ± 157	766 ± 201
CFI, per min	3.9 ± 1.5	4.0 ± 1.5	4.4 ± 1.2	4.6 ± 1.4
SVRI, dynes/sec/cm^5^/m^2^	1,654 ± 639	1,993 ± 709^a^	1,523 ± 355	2,057 ± 527^a^

CFI, cardiac function index; CI, cardiac index; DAP, diastolic arterial pressure; GEDVI, global end-diastolic volume index; HR, heart rate; MAP, mean arterial pressure; NE, norepinephrine; SVI, stroke volume index; SVRI, index of systemic vascular resistance.

^a^*P *< 0.05 vs. before norepinephrine.

## Discussion

The main result of this study performed during life-threatening septic shock is that early administration of norepinephrine aimed at restoring a sufficient MAP was associated with increases in CI and SVI, which were likely related to increases in both cardiac preload and contractility. In patients with cardiac systolic dysfunction, norepinephrine exerted either beneficial or neutral but not deleterious hemodynamic effects.

One of the striking effects of norepinephrine in our patients was its cardiac preload effect. In our whole population, norepinephrine significantly increased CI, SVI, and GEDVI, which is considered a measure of cardiac preload [15,16]. Moreover, norepinephrine significantly reduced SVV, which is a marker of volume responsiveness [20]. This result was in line with the reduction in pulse-pressure variation with norepinephrine reported by Sennoun *et al. *[21] in animals after endotoxin administration. Such findings could be ascribed to the α-mediated effect of reduction in systemic venous capacitance [4]. This hypothesis is in agreement with a previous clinical study that reported an elevation of the central blood volume--measured by transpulmonary thermodilution--in response to an acute norepinephrine-induced increase in systemic vascular resistance [22]. In our study, two findings suggest that many of our patients still had some preload reserve before norepinephrine introduction/increase: (a) the fact that GEDVI and CI both increased, and (b) the fact that the mean baseline SVV was above the presumed threshold value detecting volume responsiveness [20], and this in despite a rather low tidal volume. This was quite expected because norepinephrine infusion was started early, on the basis of a life-threatening hypotension, defined by a low diastolic arterial pressure ≤40 mm Hg and not after that volume resuscitation had been completed, as recommended by the French consensus conference [14]. Because we included many patients with prior hypertension, we sometimes introduced norepinephrine (or increased its dose) while the diastolic arterial pressure was between 40 and 55 mm Hg. The dose of norepinephrine was left to the discretion of our attending physicians, who are used to targeting an MAP value ≥65 mm Hg in patients with no history of hypertension and generally more than 75 mm Hg in the elderly or in patients with history of hypertension. This clinical practice was quite in agreement with current recommendations [1,14]. The findings of this observational study show that these goals were fairly well achieved. The achieved value of MAP was <75 mm Hg in one half of the population and ≥75 mm Hg and in the other half. It must be stressed that only 16% of patients had an achieved MAP ≥85 mm Hg, a value that is generally considered the average MAP of the normal population [23].

In our whole population, norepinephrine also increased CFI, which is considered a global index of cardiac systolic function [17-19] and thus is assumed to depend on both contractility and afterload. In the face of the increase in MAP and hence in left ventricular afterload, the increase in CFI induced by norepinephrine suggests that this drug actually increased the cardiac contractility. By using fast-response thermodilution, Martin *et al. *[24] reported an improved right ventricular function in septic-shock patients treated with norepinephrine and attributed this beneficial effect to the β_1_-agonist effect of the drug or to its ability to increase the coronary perfusion pressure by correcting systemic hypotension or both. These two mechanisms were likely to have occurred in our patients. First, it is expectable that the sepsis-induced decreased responsiveness of the myocardium to β-adrenergic stimulation [25] was not yet complete in our patients, because they were studied at a very early stage of their disease. Second, because our patients either did not receive catecholamine before inclusion or received norepinephrine only a short time before inclusion, it is unlikely that downregulation of β_1_-adrenergic receptors occurred through a tachyphylaxis mechanism [26]. In two recent studies performed in patients with septic shock who received norepinephrine after optimization of preload, norepinephrine increased CI and SVI while the achieved MAP value was ≥85 mm Hg [27,28]. Their results suggest that norepinephrine could have exerted a positive inotropic effect.

In our study, we globally found the same results in the group of 71 patients with a baseline LVEF >45% than in the whole population. Even in the group of 34 patients with a baseline LVEF ≤45%, norepinephrine increased SVI and CI. These results must be underlined because an increase in left ventricular afterload has the potential to reduce SVI in cases of impaired cardiac contractility. It is likely that the preload effect of norepinephrine was important in these patients, who probably still had some cardiac preload reserve. However, in the subset of 17 patients with both a baseline LVEF ≤45% and an achieved MAP ≥75 mm Hg, norepinephrine introduction/increase did not change SVI and CI. It is possible that in these patients, a "negative" afterload effect counterbalanced the beneficial effects of this agent. Whether targeting a lower MAP would have resulted in an increased SVI in this particular category of patients was not tested in our study.

Our study has some limitations. Because of the observational nature of the study, we were able to analyze only time points when the achieved MAP value was ≥65 mm Hg. We could not thus ascertain that norepinephrine exerts positive hemodynamic effects when a MAP <65 mm Hg is reached. Half of our patients had an achieved MAP ≥75 mm Hg, a value that can be judged relatively high, although it was still far lower than the average MAP of the normal population (>85 mm Hg) [23]. However, our studied population included many old patients or patients with preexisting comorbidities and particularly preexisting hypertension, or both. Under these conditions, the international guidelines currently recommend to target MAP at a higher level than that in patients without comorbidities [1]. In addition, it must be remembered that in the study by Rivers *et al. *[13], the MAP actually reached an average value of 95 mm Hg after 6 hours of hemodynamic treatment in the protocol group, whereas it was initially planned to target the MAP between 65 and 90 mm Hg. We used an LVEF value of 45% as a threshold to define cardiac systolic dysfunction, as did other investigators [29]. In the presence of low MAP and hence of low afterload, an LVEF ≤45% is presumed to be indicative of a poor left ventricular contractility. We evaluated the short-term effect of norepinephrine on hemodynamics and thus cannot exclude different effects of norepinephrine after a longer period. We studied septic-shock patients with life-threatening hypotension requiring early administration of norepinephrine, regardless the degree of prior volume resuscitation. Consequently, we cannot extrapolate our results to sepsis patients with no or less-severe hypotension -- as those studied by Rivers *et al. *[13] -- that are generally fully volume resuscitated before receiving norepinephrine. The fact that norepinephrine is able to improve hemodynamics, mainly through a preload effect during the early life-threatening phase, cannot imply that norepinephrine should be used instead of fluid at later periods. It is rather generally advised to make efforts to reduce the dose of the vasopressor as soon as possible [1], with the possibility of infusing fluid in case of unmasked cardiac preload reserve.

The aim of resuscitation is not only to correct hypotension but also to improve tissue oxygenation. During experimental endotoxic shock, Sennoun *et al. *[21] showed that early administration of norepinephrine in addition to fluid infusion resulted in a lower lactate level than in the case of fluid infusion alone. In our study, because we did not examine the changes in blood lactate levels, we cannot ensure that tissue oxygenation also improved after the MAP has been corrected.

## Conclusions

During septic shock with life-threatening hypotension, early administration of norepinephrine aimed at achieving a sufficient perfusion pressure is also able to increase cardiac output through increases in cardiac preload and contractility. Such a beneficial effect remained significant in patients with poor cardiac contractility, except when MAP values ≥75 mm Hg were achieved.

## Key messages

• Early administration of norepinephrine aimed at rapidly achieving a sufficient perfusion pressure in severely hypotensive septic-shock patients is able to increase cardiac output.

• The increase in cardiac output with norepinephrine seems to be related to increases in both cardiac preload and cardiac contractility.

• These positive effects can be observed even in patients with poor cardiac contractility, except when values of MAP ≥75 mm Hg are achieved.

## Abbreviations

CFI: cardiac function index; CI: cardiac index; GEDVI: global end-diastolic volume index; ICU: intensive care unit; LVEF: left ventricular ejection fraction; MAP: mean arterial pressure; SVI: stroke volume index; SVRI: index of systemic vascular resistance.

## Competing interests

Professors Jean-Louis Teboul and Xavier Monnet are members of the medical advisory board of Pulsion Medical Systems (Germany). All other authors declare that they have no competing interests.

## Authors' contributions

OH participated in study design and in data collection and interpretation, performed the statistical analysis, and drafted the manuscript. JFG, XM, HK, and JM participated in study design and data collection. CR participated in data interpretation. JLT conceived the study and its design and helped to draft the manuscript. All the authors read and approved the final manuscript.
